# The Role of Photodynamic Therapy Mediated by Natural Photosensitisers in the Management of Peri-Implantitis: A Systematic Review

**DOI:** 10.3390/pharmaceutics17040443

**Published:** 2025-03-30

**Authors:** Aleksandra Warakomska, Jakub Fiegler-Rudol, Magdalena Kubizna, Dariusz Skaba, Rafał Wiench

**Affiliations:** 1 Department of Periodontal Diseases and Oral Mucosa Diseases, Faculty of Medical Sciences in Zabrze, Medical University of Silesia, 40-055 Katowice, Poland; awarakomska@sum.edu.pl (A.W.); dskaba@sum.edu.pl (D.S.); 2 Department of Oral Surgery, Faculty of Medical Sciences in Zabrze, Medical University of Silesia, 40-055 Katowice, Poland; mkubizna@acstom.bytom.pl

**Keywords:** peri-implantitis, photodynamic therapy, curcumin, riboflavin, 5-ALA, antimicrobial therapy, dental implants, biofilm

## Abstract

**Background**: Peri-implantitis, an inflammatory condition leading to progressive bone loss around dental implants, represents a significant challenge in modern implant dentistry. Conventional mechanical debridement and adjunctive antibiotics or antiseptics often fail to fully eradicate complex biofilms and may promote antibiotic resistance. Photodynamic therapy (PDT) mediated by natural photosensitizers (e.g., curcumin, riboflavin, and 5-aminolevulinic acid) has emerged as a potential adjunctive strategy for peri-implantitis management due to its targeted antimicrobial and anti-inflammatory effects. **Objectives**: This systematic review aimed to evaluate the antimicrobial efficacy, clinical outcomes, and safety of PDT mediated by natural photosensitizers in the treatment of peri-implantitis and to identify optimal protocols regarding photosensitizer concentrations, light source parameters, and application techniques. Methods: Following PRISMA 2020 guidelines, databases (PubMed/Medline, Embase, Scopus, and Cochrane Library) were searched from 1 January 2015 to 3 January 2025 for English-language publications. Studies assessing naturally based PDT interventions for peri-implantitis or in vitro biofilms from diseased implant surfaces were included. Quality assessment used the Revised Cochrane Risk of Bias tool (RoB 2) for randomized controlled trials and a tailored nine-item framework for in vitro studies. Eleven studies met the inclusion criteria. **Results**: Despite heterogeneity in methodologies, especially regarding light wavelengths, energy densities, and photosensitizer formulations, most studies reported notable reductions in bacterial viability, biofilm mass, and clinical indices (probing depth and bleeding on probing). Curcumin and riboflavin frequently demonstrated comparable antimicrobial efficacy to standard disinfectants, while 5-aminolevulinic acid (5-ALA)-based PDT also showed promising clinical and microbiological improvements. However, complete biofilm eradication was rarely achieved. **Conclusions**: Natural-photosensitizer-based PDT appears to be a valuable adjunct to mechanical debridement for peri-implantitis, enhancing microbial control and clinical outcomes. Standardization of PDT protocols and further well-designed clinical trials with extended follow-up periods are warranted to confirm long-term efficacy and inform evidence-based guidelines.

## 1. Introduction

### 1.1. Rationale

Peri-implant diseases are inflammatory conditions around dental implants, such as peri-implant mucositis and peri-implantitis [1]. They involve inflammation that may lead to supporting bone loss [2] and are a growing concern in implant dentistry [1,2]. Peri-implant mucositis is a reversible condition marked by redness, swelling, and bleeding on probing without further bone loss [3], whereas peri-implantitis is irreversible, showing similar inflammatory signs with progressive bone loss and increased probing depths [2,4,5,6,7,8,9]. This progression parallels the transition from gingivitis to periodontitis in natural teeth, highlighting the need for early intervention [2,3,4,5,6,7,8,9]. The primary etiological factor is the oral biofilm [10]; however, systemic and local factors, including diabetes mellitus, tobacco use, genetic predisposition, insufficient keratinized gingiva, poor oral hygiene, and prosthetic design flaws, also play a role [11,12]. Furthermore, metallosis from titanium particles and ions released due to tribocorrosion and fretting can elicit an inflammatory response that exacerbates bone loss [13,14,15,16,17]. Management typically relies on mechanical debridement to disrupt biofilms [18,19,20], but this method often fails to eradicate biofilm from the implant’s surface. Adjunctive antiseptics and antibiotics have been used to improve outcomes; however, they are associated with antibiotic resistance, adverse side effects, and limited long-term efficacy [19,20,21]. Consequently, alternative strategies are being explored, such as photodynamic therapy (PDT), which uses light, a photosensitizer, and oxygen to generate reactive oxygen species that destroy microbial cells [22,23]. PDT’s distinct mechanism reduces resistance risk and allows for targeted microbial killing [24]. Recent attention has focused on natural photosensitizers like curcumin and riboflavin for antimicrobial photodynamic therapy (aPDT) [25,26]. These agents are biocompatible, exhibit broad-spectrum antimicrobial activity and possess anti-inflammatory and antioxidant properties, making them suitable for managing peri-implant diseases [22,23,24,25,26]. Curcumin, a polyphenol, disrupts biofilm integrity and has potent antimicrobial effects [26,27], while riboflavin, upon light activation, produces singlet oxygen and other reactive oxygen species that target bacterial membranes and DNA [25,28,29]. Both also promote wound healing and reduce inflammation, aiding in the management of peri-implant tissue damage [28,29].

### 1.2. Objectives

The primary objective of this systematic review is to evaluate the clinical efficacy, antimicrobial potential, and safety of aPDT mediated by natural photosensitisers in the management of peri-implantitis. Additionally, it seeks to identify optimal treatment protocols, including light source parameters, photosensitizer concentrations, and application techniques, to provide evidence-based recommendations for clinical use. By addressing these aspects, this review intends to bridge the gap between experimental findings and practical applications, offering insights into the potential of these natural photosensitizers as adjunctive therapies in peri-implantitis management.

## 2. Materials and Methods

### 2.1. Focused Question

A systematic review was conducted following the PICO framework [30], structured as follows: in patients with clinically diagnosed peri-implantitis or ex vivo specimens (biofilms) derived from the diseased implant surfaces—reflecting the true polymicrobial biofilm complexity observed in peri-implantitis (population), does treatment with antimicrobial photodynamic therapy mediated by natural photosensitisers (intervention), compared to traditional mechanical debridement, the use of natural photosensitisers alone, or other non-surgical therapies (comparison), result in more effective management or reduction of peri-implantitis symptoms (outcome)?

### 2.2. Search Strategy

**Figure 1:**
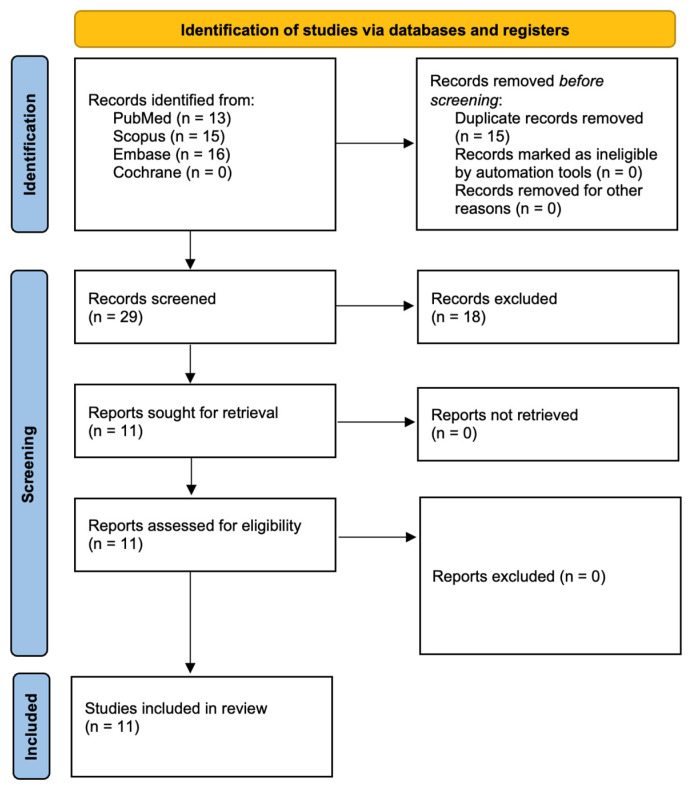
Prisma 2020 flow diagram.

This systematic review was registered with PROSPERO under the registration number CRD42025635967. The review process was aligned with the PRISMA 2020 (Preferred Reporting Items for Systematic Reviews and Meta-Analyses) guidelines to ensure comprehensive and transparent reporting [31]. An extensive literature search was executed across several databases, including PubMed/Medline, Embase, Scopus, and the Cochrane Library, using the specific search terms detailed in Table 1. Three researchers independently conducted the database searches employing identical search strategies to maintain consistency. To refine the search results, inclusion criteria were set to encompass studies published from 1 January 2015 to 3 January 2025 and limited to articles published in English. Following the initial retrieval, studies were screened based on their titles and abstracts to ascertain their relevance and adherence to the inclusion criteria outlined in Section 2.3. Subsequently, two authors performed a comprehensive full-text assessment of the shortlisted articles to extract pertinent data systematically. In addition to the primary search, a snowballing technique was employed by reviewing the reference lists of eligible studies to identify any further relevant research that might have been missed initially. This review proposes that photodynamic therapy utilizing natural photosensitizers may offer a superior or supplementary approach to the management of peri-implantitis, potentially enhancing treatment outcomes compared to traditional non-surgical interventions. Studies incorporated into this analysis were meticulously selected based on defined inclusion and exclusion criteria to ensure the relevance and quality of the evidence gathered. Figure 1 shows the selection process.

### 2.3. Selection of Studies

In the selection phase of this systematic review, each reviewer individually assessed the titles and abstracts of the retrieved articles to ensure impartiality. When disagreements arose concerning a study’s eligibility, the reviewers engaged in collective discussions until they reached unanimous consensus. This meticulous approach, in line with PRISMA guidelines, guaranteed that only the most relevant and high-quality studies were incorporated into the analysis, thereby enhancing the review’s reliability and reproducibility [31]. The following inclusion and exclusion were used to decide which studies to include.

Inclusion criteria are shown as follows:

- Investigations utilizing natural photosensitizers in photodynamic therapy for the treatment of peri-implantitis, encompassing both in vitro and animal studies.
- Randomized controlled trials where natural photosensitizers serve as the primary photosensitizer in PDT for managing peri-implantitis.
- Research evaluating the combined effects of natural photosensitizers in PDT with other antimicrobial or anti-inflammatory treatments.
- Studies incorporating control groups that compare aPDT mediated by natural photosensitisers against standard mechanical debridement, alternative non-surgical therapies, or no treatment.
- Studies directly comparing the effectiveness of aPDT mediated by natural photosensitisers with other non-surgical treatment modalities for peri-implantitis.
- Research featuring extended follow-up periods to assess the sustained impact of naturally mediated aPDT on peri-implantitis management.
- Articles that meet predefined quality standards and specifically address the reduction or management of peri-implantitis symptoms using naturally mediated aPDT.

Exclusion criteria are shown as follows:

- Unpublished theses, conference abstracts, dissertations, and other non-peer-reviewed materials.
- Articles published in languages other than English.
- No full text available.
- Multiple reports of the same study or those sharing identical ethical approval numbers.
- Research focused on dental or medical issues unrelated to peri-implantitis.
- Studies utilizing synthetic photosensitizing agents.
- Laboratory studies that do not replicate oral conditions pertinent to peri-implantitis or fail to address relevant microbial strains.
- Case reports, case series, narrative reviews, systematic reviews, editorials, books, and other formats that do not provide original research data.
- Studies that do not include a control or comparison group.
- Applications of PDT not intended as a therapeutic method for peri-implantitis.

Limiting the review to English-language publications and excluding the grey literature was a deliberate choice to ensure methodological rigor and manage resource constraints, as English-language studies typically represent the bulk of high-quality, peer-reviewed research and the grey literature often lacks a comprehensive peer review needed for reliable data extraction. This approach enhances the consistency, reproducibility, and overall reliability of the review, though it may introduce language and publication biases by potentially overlooking relevant studies published in other languages or in non-peer-reviewed formats.

### 2.4. Data Extraction

At the beginning of the study selection, each reviewer separately evaluated the titles and abstracts of the retrieved articles to reduce the risk of bias. The agreement between reviewers was quantified using Cohen’s kappa statistic to ensure consistent decision-making [32]. In cases where reviewers disagreed on the eligibility of a study, thorough discussions were conducted among the authors until a unanimous consensus was reached. After finalizing the selection of relevant studies, the three reviewers systematically extracted comprehensive data from each included article. The data encompassed bibliographic information such as the first author and publication year, study design, specific peri-implantitis cases addressed, and the types of test and control groups used. Additionally, they recorded the duration of follow-up periods and measured outcomes related to peri-implantitis management. They detailed the specifications of the light sources employed, including type, wavelength, and power parameters. Information on the concentrations of photosensitizers, the incorporation of nanocarriers or other adjunctive substances, as well as the incubation and irradiation times during photodynamic therapy, was also meticulously documented. This thorough data extraction process ensured that all relevant variables were captured, enabling a robust and comprehensive analysis of the efficacy and parameters of naturally mediated photodynamic therapy in the treatment of peri-implantitis. Our systematic review identified significant clinical heterogeneity among studies evaluating naturally mediated photodynamic therapy for peri-implantitis. Variability in photosensitizer concentrations, light source parameters, incubation times, and study designs complicates direct comparisons and efficacy interpretation. Standardized protocols and further high-quality trials are needed to optimize aPDT for clinical use. Outcome measurements in this systematic review primarily include microbial load reduction (colony-forming units per milliliter, CFU/mL), biofilm mass reduction (crystal violet assay, scanning electron microscopy), changes in microbial viability and metabolic activity, reactive oxygen species (ROS) generation, downregulation of biofilm-related genes, and clinical improvements such as bleeding on probing (BoP), probing depth (PD), plaque index (PI), and marginal bone level (MBL), providing a comprehensive evaluation of the efficacy of naturally mediated photodynamic therapy in managing peri-implantitis.

## 3. Results

### 3.1. Study Selection

Figure 1 presents the comprehensive research process aligned with the PRISMA guidelines [31]. The initial literature search identified 44 articles, which were subsequently reduced to 29 after removing duplicates. Upon reviewing the titles and abstracts, 11 studies were selected for full-text assessment. 2 Studies were excluded, as they were reviews [33,34]. Ultimately, 11 studies were included in the final analysis. 

### 3.2. Quality Assessment Presentation and Risk of Bias in Individual Studies

For clinical studies, the quality assessment was conducted using the Revised Cochrane Risk of Bias Tool (RoB 2) for randomized controlled trials (RCTs) [35,36]. We applied the RoB2 by evaluating key domains—randomization, deviations from intended interventions, missing outcome data, outcome measurement, and selective reporting—assigning ratings of low risk, some concerns, or high risk to inform our overall assessment. For the in vitro studies, we developed a tailored framework based on nine essential criteria: clear specification of photosensitizer concentration and origin, defined incubation period, detailed light source parameters (wavelength, power, fluence, and power density), use of a calibrated power meter, inclusion of a negative control group, presentation of statistical analyses, completeness of outcome data, and independence from funding sources. These criteria were chosen to ensure methodological consistency, reproducibility, and reliability of findings, thereby strengthening the rigor of our review. The results of this test is shown in Table 2.

The methodological quality of the other study types was independently evaluated by three reviewers, focusing on key aspects of PDT protocols, including study design, implementation, and data analysis, to ensure objectivity and result reliability. Potential biases were identified by assigning a score of 1 for each affirmative (“yes”) response and 0 for each negative (“no”) response based on predefined evaluation criteria: (1) specification of photosensitizer concentration, (2) disclosure of the photosensitizer’s origin or source, (3) clear definition of the incubation period, (4) provision of detailed light source specifications (type, wavelength, output power, fluence, and power density), (5) use of a power meter to measure light parameters, (6) inclusion of a negative control group in the experimental design, (7) presentation of numerical results with relevant statistical analyses, (8) absence of missing data regarding study outcomes, and (9) independence of the study from its funding sources. Extracted data were systematically analyzed and categorized based on the total number of affirmative responses, with the risk of bias classified as high (0–3 points), moderate (4–6 points), or low (7–9 points). Each study’s total score determined its corresponding bias risk level according to the guidelines in *The Cochrane Handbook for Systematic Reviews of Interventions* [38]. This rigorous quality assessment ensured that only studies with robust and reliable methodologies were included in the systematic review, thereby enhancing the overall validity and credibility of the findings. The results of this quality assessment are presented in Table 3.

### 3.3. Data Presentation

Table 4, Table 5, Table 6, Table 7, Table 8, Table 9, Table 10 and Table 11 present a comprehensive summary of the data extracted from the eight studies that satisfied the inclusion criteria and were incorporated into the review. The tables detail the general characteristics of the studies, the properties of the light sources utilized, and the specific features of employed photosensitizers in aPDT protocols.

### 3.4. General Characteristics of the Included Studies

Table 4 outlines the general characteristics of the eight studies included in the analysis.

### 3.5. Characteristics of Light Sources Used in aPDT

Table 6 provides details on the physical parameters of the light sources utilized in the studies meeting the inclusion criteria.

The significant heterogeneity in photodynamic therapy protocols—including variations in light wavelength, energy density, photosensitizer concentration, and incubation time—poses a challenge in drawing definitive conclusions from the aggregated data. To address this, we meticulously extracted and tabulated key methodological parameters from each study, enabling us to identify both commonalities and critical differences. We then employed a narrative synthesis. Additionally, rigorous quality assessments allowed us to weight findings from studies with more standardized protocols more heavily. Ultimately, while our findings support the potential of naturally mediated aPDT as promising adjunctive treatments for peri-implantitis, the observed variability underscores the need for standardized protocols and further research to ensure more precise, comparable, and clinically translatable results.

## 4. Discussion

### 4.1. Results in the Context of Other Evidence

The systematic review highlights the promising potential of naturally mediated aPDT in managing peri-implantitis, offering significant antimicrobial efficacy and clinical benefits [38,39,40,41,42,43,44,45,46,47]. Curcumin-mediated aPDT, activated by LED light, demonstrated comparable antimicrobial properties to chlorhexidine, the gold standard for implant decontamination [38]. Riboflavin-aPDT, activated by blue LED, effectively reduced Staphylococcus aureus biofilms with aesthetic advantages due to its light-yellow colour [39]. Cur-aPDT also showed significant impacts on quorum sensing and bystander effects, enhancing its overall efficacy against *Aggregatibacter actinomycetemcomitans* [40]. Riboflavin-based PDT with a 445 nm laser reduced biofilm on titanium surfaces, proving particularly suitable for aesthetic zones [41]. Graphene quantum dots combined with curcumin enhanced ROS production and antimicrobial activity [42]. Riboflavin-loaded nanoparticles in aloe vera gel significantly improved clinical parameters in peri-implantitis treatment, particularly in diabetic patients [43]. Curcumin-loaded nanoparticles showed high efficacy against planktonic bacteria but limited effects on biofilms [44]. However, curcumin/DMSO-based aPDT revealed limited antibacterial effects when combined with a 445 nm laser, emphasizing the need for optimized protocols [45]. Additionally, three studies evaluating 5-ALA-mediated photodynamic therapy (5-ALA PDT) have underscored its potential in peri-implantitis management [46,47,48]. Rossi et al. reported that applying a 5% 5-ALA gel significantly reduced probing pocket depth and bleeding on probing at 3 and 6 months post-treatment, showing promise as an adjunctive therapy [46]. Petrini et al. demonstrated that 5-ALA-based PDT effectively curtailed *Streptococcus oralis* biofilm formation on titanium surfaces [47]. Similarly, Radunović et al. found that 5-ALA gel, particularly at higher concentrations, exerted a strong bactericidal effect against multiple bacterial strains relevant to peri-implantitis [48]. These findings collectively reinforce the potential role of 5-ALA-mediated PDT in peri-implant disease management, paralleling the encouraging outcomes reported for curcumin- and riboflavin-mediated approaches. Despite the promising results, significant variability in light source parameters, photosensitizer concentrations, and incubation times across studies hinders the development of standardized guidelines [38,39,40,41,42,43,44,45,46,47]. PDT consistently demonstrated efficacy in biofilm reduction, but complete eradication was rarely achieved, highlighting its role as an adjunctive rather than a standalone therapy [38,39,40,41,42,43,44,45,46,47]. These findings underscore the potential of aPDT in peri-implantitis management while emphasizing the need for further standardization and clinical trials to establish optimized protocols and confirm long-term efficacy [38,39,40,41,42,43,44,45,46,47]. Similar research has shown comparable observations. For instance, Rahman et al. concluded that aPDT reduces bacterial loads associated with peri-implant diseases and may serve as an alternative to antibiotics, offering short-term benefits as an adjunct to mechanical debridement [49], though its clinical relevance requires further investigation and careful case selection [49]. Likewise, Fraga et al. demonstrated significant reductions in bacterial counts of *Aggregatibacter actinomycetemcomitans*, *Porphyromonas gingivalis*, and *Prevotella intermedia*, underlining PDT’s efficacy in reducing microbial loads in peri-implantitis [50]. Lopez et al. reported improvements in bleeding on probing, probing depth, clinical attachment level, plaque index, gingival index, and bone level density after PDT treatment [51,52,53,54]. Zhao et al. showed that PDT and antibiotics, used adjunctively, were both effective in reducing bacterial loads [55,56,57]. Joshi et al. confirmed notable benefits from PDT as a non-surgical peri-implantitis management strategy [58,59,60,61]. Although PDT has demonstrated significant positive outcomes, no clear superiority of laser-based therapies over conventional peri-implantitis interventions has been definitively established, given the heterogeneity in study designs and laser parameters. Bombeccari et al. found that while PDT reduced bleeding and inflammatory exudation in peri-implantitis patients, it did not notably decrease total anaerobic bacteria compared to surgical therapy at 24 weeks [62,63,64,65]. Schär et al. reported that non-surgical mechanical debridement with adjunctive PDT was as effective as local drug delivery (minocycline microspheres) in reducing mucosal inflammation over 6 months, although neither modality consistently achieved complete resolution [66,67,68]. In another study, Wang et al. observed that aPDT combined with mechanical debridement significantly improved periodontal parameters in peri-implantitis patients over 6 months, surpassing mechanical debridement alone in improving clinical attachment loss [69]. Thus, aPDT shows promise as an adjunctive peri-implantitis therapy, particularly for reducing inflammation and improving clinical outcomes, though its efficacy is influenced by treatment protocols and comparative modalities [49,50,51,52,53,54,55,56,57,58,59,60,61,62,63,64,65,66,67,68,69].

### 4.2. Other Natural Photosensitisers Potentially Appropriate for aPDT Against Periimplantitis

In addition to widely studied natural compounds such as curcumin, riboflavin, and 5-aminolevulinic acid, several other naturally derived photosensitizers merit attention for their potential role in antimicrobial photodynamic therapy targeting peri-implantitis [70,71]. These compounds not only expand the photochemical toolbox but also align with the growing preference for biocompatible and plant-based therapeutic agents [71]. Among them, hypericin, derived from Hypericum perforatum (St. John’s Wort), has emerged as a potent photosensitizer owing to its strong absorption in the visible spectrum and exceptional ability to generate singlet oxygen [72,73]. Beyond its antimicrobial capacity, hypericin also exhibits notable anti-inflammatory properties, a dual functionality that could be particularly advantageous in the inflammatory milieu of peri-implant diseases [25,74]. Chlorophyllin, a semi-synthetic, water-soluble derivative of chlorophyll, is another promising candidate [75,76,77]. It absorbs efficiently within the therapeutic window (630–700 nm), enabling effective light activation with standard diode lasers used in dental settings [78]. Notably, chlorophyllin has demonstrated antimicrobial efficacy against both planktonic and biofilm-associated pathogens, an essential requirement in managing the complex biofilm structures on implant surfaces [79,80]. Protoporphyrin IX, an endogenous precursor in the heme biosynthesis pathway, has long been recognized for its high quantum yield in reactive oxygen species (ROS) generation [81,82]. Already used clinically in photodynamic diagnosis and treatment of neoplastic lesions, its potential for application in peri-implantitis warrants further investigation, particularly considering its intrinsic compatibility with host tissues [83]. In addition, metal-substituted phthalocyanines, such as zinc (ZnPc), aluminum (AlPc), and aluminum chloride (AlClPc) derivatives, have gained attention due to their strong absorption in the red to near-infrared spectrum (600–800 nm), which allows deeper tissue penetration [84,85]. Their excellent photostability, high singlet oxygen quantum yields, and tunable chemical structure make them compelling candidates for targeted aPDT approaches in peri-implant environments [86,87]. Although these agents were not the primary focus of this review due to the limited volume of direct evidence pertaining specifically to peri-implantitis, their well-documented photophysical properties, established antimicrobial profiles, and favorable safety data underscore their relevance. As such, their inclusion in future experimental and clinical studies may significantly broaden the scope of naturally mediated aPDT. Incorporating these compounds could enhance therapeutic flexibility, improve patient outcomes and contribute to the development of more sustainable, nature-inspired treatment strategies in peri-implant disease management.

### 4.3. Limitations of the Evidence

The evidence presented in this systematic review is subject to several limitations that must be acknowledged. One of the most significant challenges is the heterogeneity among the included studies, particularly regarding variations in aPDT protocols, such as light source parameters (wavelength, energy density, and fluence), photosensitizer concentrations, and incubation times. These discrepancies hinder direct comparisons between studies and limit the ability to develop standardized treatment protocols. Additionally, many studies relied heavily on in vitro models, which, while valuable, do not fully replicate the complex biological environment of peri-implantitis in vivo. The limited number of clinical trials and their relatively short follow-up periods further restrict the generalizability and long-term applicability of the findings. Furthermore, the studies often used subjective measures, such as CFU reduction or visual biofilm assessments, without integrating advanced diagnostic tools like molecular analyses or imaging techniques to provide a deeper understanding of treatment efficacy. Lastly, the exclusion of non-English articles and the grey literature may have introduced a selection bias, potentially overlooking relevant data that could have enriched the review’s findings. Addressing these limitations in future research is crucial for advancing the clinical utility of PDT in peri-implantitis management.

### 4.4. Limitations of the Review Process

The review process faced several limitations that impacted its scope and generalizability. Significant heterogeneity among the included studies, particularly in photodynamic therapy protocols such as light parameters, photosensitizer concentrations, and incubation times, hindered the ability to conduct a meta-analysis and required a narrative synthesis of results. Language restrictions excluded non-English studies, potentially omitting valuable data, while the exclusion of the grey literature, such as conference abstracts and unpublished research, may have introduced publication bias. Some studies lacked detailed reporting of methodological aspects, including light settings and incubation periods, complicating assessments of replicability and reliability. The focus on recent publications from 2014 to 2024, while ensuring contemporary relevance, might have excluded earlier foundational studies. Although data extraction and study selection were independently performed by multiple reviewers, the resolution of disagreements through discussion introduced some subjectivity. Furthermore, the lack of uniform application of standardized quality assessment tools, such as the GRADE framework, and the reliance on in vitro studies limited the clinical applicability of the findings. Additionally, many studies featured short follow-up periods, restricting the evaluation of long-term PDT efficacy.

### 4.5. Implications for Practice, Policy, and Future Research

In clinical practice, curcumin- and riboflavin-mediated aPDT shows promise as a safe and effective adjunctive treatment, offering an alternative to conventional mechanical debridement and antibiotics, particularly for biofilm-associated infections. Clinicians should consider incorporating aPDT into treatment protocols, especially in cases where traditional methods are insufficient or contraindicated. However, the lack of standardized treatment parameters, including light source characteristics, photosensitizer concentrations, and application techniques, underscores the need for clear clinical guidelines. Policymakers and professional organizations should prioritize the development and dissemination of evidence-based protocols and support training programs to facilitate the integration of aPDT into routine dental practice. Future research should focus on addressing the gaps identified in this review, including the variability in aPDT protocols and the limited number of high-quality clinical trials. Large-scale, multicenter randomized controlled trials with standardized parameters are essential to establish the long-term efficacy and safety of aPDT in peri-implantitis management. Additionally, exploring the synergistic effects of aPDT combined with other therapeutic modalities, such as antimicrobial agents or nanocarriers, may enhance treatment outcomes. Innovations in photosensitizer delivery systems, such as hydrogels or nanoparticles, should also be investigated to improve biofilm penetration and stability. Furthermore, advanced diagnostic tools, including imaging and molecular techniques, should be integrated into studies to provide more objective outcome measures. These efforts will help bridge the gap between experimental findings and practical applications, ultimately advancing the role of aPDT in peri-implant disease management and improving patient outcomes. Clinicians may integrate PDT with conventional therapies, such as mechanical debridement and antiseptic or antibiotic treatments, to enhance microbial reduction and biofilm disruption, thereby potentially improving clinical outcomes. This combined approach leverages PDT’s benefits, such as its non-invasive nature, reduced risk of antibiotic resistance, and anti-inflammatory effects, while compensating for its limitations in completely eradicating biofilms on its own. However, the variability in PDT protocols underscores the need for standardized parameters to ensure consistent efficacy. In practice, this means that while PDT can bolster current treatment protocols, clinicians must carefully evaluate patient-specific factors and adhere to optimized, evidence-based protocols to achieve the best long-term results.

Our review identifies several key gaps in the current literature that warrant further investigation. First, there is significant variability in treatment parameters, including photosensitizer type and concentration, light wavelength, energy density, and incubation time, which hampers direct comparisons and calls for the development of standardized protocols. Second, the limited number of high-quality, long-term clinical trials restricts our ability to fully evaluate the efficacy and safety of photodynamic therapy as an adjunctive treatment. Future research should aim to harmonize these critical variables and implement objective outcome measures, while also exploring advanced delivery systems (e.g., nanoparticle carriers) and potential synergistic effects when PDT is combined with conventional therapies. Addressing these gaps through multicenter randomized controlled trials will be essential to refine clinical protocols and fully establish the role of PDT in managing peri-implantitis.

## 5. Conclusions

This systematic review shows that photodynamic therapy mediated by natural photosensitizers, particularly curcumin, riboflavin, and 5-aminolevulinic acid (5-ALA), can significantly reduce microbial loads, disrupt biofilms and improve clinical parameters in peri-implantitis. Although the findings are promising, heterogeneity in photosensitizer concentrations, light source parameters, and irradiation protocols complicates direct comparisons across studies. Additional large-scale, rigorously designed clinical trials are necessary to determine optimal treatment conditions, confirm long-term efficacy and facilitate the development of standardized guidelines. Natural-photosensitizer-based photodynamic therapy, including 5-ALA, holds potential as an effective adjunctive treatment strategy, especially in cases where conventional mechanical debridement and antibiotic therapy have shown limited success.

## Figures and Tables

**Table 1:** Search syntax used in the study.

Source	Search Term	Number of Results
Medline via PubMed	((“Curcumin”[MeSH] OR “Curcumin”) OR (“Riboflavin”[MeSH] OR “Riboflavin” OR “Vitamin B2”) OR (“Hypericin”[MeSH] OR “Hypericin”) OR (“Chlorophyll”[MeSH] OR “Chlorophyll” OR “Chlorophyllin”) OR (“Carotenoids”[MeSH] OR “Carotenoids” OR “Lycopene”) OR (“Hematoporphyrins”[MeSH] OR “Hematoporphyrin Derivatives”) OR (“5-Aminolevulinic Acid”[MeSH] OR “5-ALA” OR “Aminolevulinic Acid”) OR (“Phthalocyanines”[MeSH] OR “Phthalocyanines” OR “ZnPc” OR “AlPc” OR “AlClPc”) OR (“Protoporphyrins”[MeSH] OR “Protoporphyrin IX” OR “Natural porphyrins”)) AND ((“Photodynamic Therapy”[MeSH] OR “Photodynamic Therapy” OR “PDT”)) AND ((“Peri-implantitis”[MeSH] OR “Peri-implantitis”))	13
Scopus	(TITLE-ABS-KEY(“Curcumin”) OR TITLE-ABS-KEY(“Riboflavin”) OR TITLE-ABS-KEY(“Vitamin B2”) OR TITLE-ABS-KEY(“Hypericin”) OR TITLE-ABS-KEY(“Chlorophyll”) OR TITLE-ABS-KEY(“Chlorophyllin”) OR TITLE-ABS-KEY(“Carotenoids”) OR TITLE-ABS-KEY(“Lycopene”) OR TITLE-ABS-KEY(“Hematoporphyrin Derivatives”) OR TITLE-ABS-KEY(“5-Aminolevulinic Acid”) OR TITLE-ABS-KEY(“5-ALA”) OR TITLE-ABS-KEY(“Aminolevulinic Acid”) OR TITLE-ABS-KEY(“Phthalocyanines”) OR TITLE-ABS-KEY(“ZnPc”) OR TITLE-ABS-KEY(“AlPc”) OR TITLE-ABS-KEY(“AlClPc”) OR TITLE-ABS-KEY(“Protoporphyrin IX”) OR TITLE-ABS-KEY(“Natural porphyrins”)) AND (TITLE-ABS-KEY(“Photodynamic Therapy”) OR TITLE-ABS-KEY(“PDT”)) AND (TITLE-ABS-KEY(“Peri-implantitis”))	15
Embase	((“curcumin”/exp OR “curcumin”) OR (“riboflavin”/exp OR “riboflavin” OR “vitamin B2”) OR (“hypericin”/exp OR “hypericin”) OR (“chlorophyll”/exp OR “chlorophyllin” OR “chlorophyll”) OR (“carotenoid”/exp OR “carotenoids” OR “lycopene”) OR (“hematoporphyrin derivative”/exp OR “hematoporphyrin derivatives”) OR (“5-aminolevulinic acid”/exp OR “5-ALA” OR “aminolevulinic acid”) OR (“phthalocyanine”/exp OR “phthalocyanines” OR “ZnPc” OR “AlPc” OR “AlClPc”) OR (“protoporphyrin”/exp OR “protoporphyrin IX” OR “natural porphyrins”)) AND (“photodynamic therapy”/exp OR “photodynamic therapy” OR “PDT”) AND (“periimplantitis”/exp OR “peri-implantitis” OR “periimplantitis”)	16
Cochrane data base	((MH “Curcumin” OR “Curcumin”) OR (MH “Riboflavin” OR “Riboflavin” OR “Vitamin B2”) OR (MH “Hypericin” OR “Hypericin”) OR (MH “Chlorophyll” OR “Chlorophyllin” OR “Chlorophyll”) OR (MH “Carotenoids” OR “Carotenoids” OR “Lycopene”) OR (MH “Hematoporphyrins” OR “Hematoporphyrin Derivatives”) OR (MH “5-Aminolevulinic Acid” OR “5-ALA” OR “Aminolevulinic Acid”) OR (MH “Phthalocyanines” OR “Phthalocyanines” OR “ZnPc” OR “AlPc” OR “AlClPc”) OR (MH “Protoporphyrins” OR “Protoporphyrin IX” OR “Natural porphyrins”)) AND (MH “Photodynamic Therapy” OR “Photodynamic Therapy” OR “PDT”) AND (MH “Peri-implantitis” OR “Peri-implantitis”)	0

**Table 2:** Results of the RoB2 test for RCTs.

Study	Study Type	Randomization Process	Deviations from Intended Interventions	Missing Outcome Data	Measurement of Outcomes	Selective Reporting	Overall RoB 2 Rating
Qamar et al., 2023 [37]	RCT	Low risk	Some concerns	Low risk	Low risk	Low risk	Some concerns

**Table 3:** The results of the quality assessment for the in vitro studies.

Author and Year	1	2	3	4	5	6	7	8	9	Total Score	Risk
Etemadi et al., 2023 [39]	1	1	1	1	0	1	1	1	0	7	Low
Leelanarathiwatat et al., 2020 [40]	1	0	1	1	0	1	1	1	1	7	Low
Mahdizade-Ari et al., 2019 [41]	1	0	1	1	1	1	1	1	1	8	Low
Morelato et al., 2022 [42]	1	1	1	1	0	1	1	1	1	8	Low
Pourhajibagher et al., 2019 [43]	1	1	1	1	1	1	1	1	1	9	Low
Tonon et al., 2022 [44]	1	0	0	1	0	1	1	1	0	5	Moderate
Wenzler et al., 2024 [45]	1	0	1	1	0	1	1	1	1	7	Low
Rossi et al., 2022 [46]	1	1	1	1	0	1	1	1	0	7	Low
Petrini et al., 2022 [47]	1	1	1	1	0	1	1	1	0	7	Low
Radunović et al., 2020 [48]	1	1	1	1	0	1	1	1	0	7	Low

**Table 4:** A general overview of the studies.

Author and Year	Country	Study Design
Qamar et al., 2023 [37]	Saudi Arabia	Clinical study
Etemadi et al., 2023 [39]	Iran and Italy	In vitro
Leelanarathiwatat et al., 2020 [40]	Thailand and Japan	In vitro
Mahdizade-Ari et al., 2019 [41]	Iran	In vitro
Morelato et al., 2022 [42]	Croatia, Bosnia, and Herzegovina	In vitro
Pourhajibagher et al., 2019 [43]	Iran and Italy	In vitro
Tonon et al., 2022 [44]	Brazil and USA	In vitro
Wenzler et al., 2024 [45]	Germany	In vitro
Rossi et al., 2022 [46]	Italy	Retrospective Analysis
Petrini et al., 2022 [47]	Italy	In vitro
Radunović et al., 2020 [48]	Serbia and Italy	In vitro

**Table 5:** Main details from RCTs.

Author and Year	Species	Photosensitizer Concentration	Outcome Measures
Qamar et al., 2023 [37]	*Porphyromonas gingivalis* and *Tannerella forsythia*	Riboflavin loaded on poly-L-glycolic acid nanoparticles incorporated in aloe vera gel (PGA/RF/AV) 0.1% riboflavin concentration in the PGA/RF/AV gel	Significant reduction in microbial counts of *P. gingivalis* and *T. forsythia* Improvement in clinical parameters: BoP, PD, PI, and MBL; Enhanced treatment outcomes compared to PDT alone.

**Table 6:** Main details from each in vitro study.

Author and Year	Species	Photosensitizer Concentration	Outcome Measures
Etemadi et al., 2023 [39]	*A. actinomycetemcomitans*	Curcumin (5 mg/mL); Riboflavin (0.5%)	CFU/mL: quantitative measure of bacterial reduction. Groups were analyzed to determine the efficacy of treatments in reducing A.a biofilm.
Leelanarathiwatat et al., 2020 [40]	*Staphylococcus aureus*	MB: 10 mg/mL FMN: 0.18 mg/mL	Log reduction in CFU/mL (colony-forming units); Biofilm mass reduction measured by crystal violet assay; Direct observation using SEM and fluorescent stereomicroscopy.
Mahdizade-Ari et al., 2019 [41]	*A. actinomycetemcomitans*	Curcumin: 80 μg/mL	Reduction in microbial cell survival; Decrease in metabolic activity; Reduction in quorum sensing; No significant change in efflux capacity.
Morelato et al., 2022 [42]	*Staphylococcus aureus* and *Candida albicans*	Riboflavin (0.1%) MB (0.1%)	Reduction in CFU of *Staphylococcus aureus* and *Candida albicans*. Antimicrobial effect was comparable between 445 nm riboflavin and 660 nm methylene blue photodynamic therapy protocols. SEM analysis showed reduced biofilm, but some microorganisms remained visible.
Pourhajibagher et al., 2019 [43]	*Aggregatibacter actinomycetemcomitans*, *Porphyromonas gingivalis*, and *Prevotella intermedia*	GQD-Cur: 100 µg/mL	Reduction in microbial cell viability; Reduction in biofilm biomass; Significant ROS generation in a dose-dependent manner; Downregulation of biofilm-related genes: rcpA (8.1-fold), fimA (9.6-fold), and inpA (11.8-fold).
Tonon et al., 2022 [44]	*Porphyromonas gingivalis*, *Fusobacterium nucleatum*, and *Streptococcus oralis*	Curcumin-loaded polymeric nanoparticles (Curcumin-NP) Concentration: 500 µg/mL	Reduction in bacterial viability in planktonic cultures (log reduction values); Limited antibiofilm activity on multispecies biofilms; Significant antimicrobial effects when photoactivated with blue light (420 nm); Non-cytotoxic to human periodontal ligament fibroblast cells.
Wenzler et al., 2024 [45]	*Aggregatibacter actinomycetemcomitans*, *Campylobacter rectus*, *Eikenella corrodens*, *Fusobacterium nucleatum*, *Porphyromonas gingivalis*, *Prevotella intermedia*, *Parvimonas micra*, *Treponema denticola*, and *Tannerella forsythia*	PDT: HELBO^®^ Blue Photosensitizer; PTT: EmunDo^®^ dye; Curcumin/DMSO solution: curcumin: 100 mg/L with 0.5% DMSO.	PDT: bacterial reduction; PTT: bacterial reduction; Curcumin/DMSO + laser: bacterial reduction.
Petrini et al., 2022 [47]	*Streptococcus oralis*	ALAD 5%	Reduction in bacterial biofilm on infected titanium surfaces
Radunović et al., 2020 [48]	*Enterococcus faecalis*, *Escherichia coli*, *Staphylococcus aureus*, *Veillonella parvula*, and *Porphyromonas gingivalis*	ALAD 10%, 25%, and 50%	Reductions in CFU

CFU—colony-forming units; MB—methylene blue; FMN—flavin mononucleotide; GQD-Cur—curcumin doped with graphene quantum dots; ROS—reactive oxygen species; BoP—bleeding on probing; PD—probing depth; PI—plaque index; MBL—marginal bone level; PDT—photodynamic therapy; PTT—photothermal therapy; SEM—scanning electron microscopy; DMSO—dimethyl sulfoxide; NP—nanoparticles; ALAD—5-aminolevulinic acid.

**Table 7:** Main details from the retrospective study.

Author and Year	Species	Photosensitizer Concentration	Outcome Measures
Rossi et al., 2022 [46]	*Porphyromonas gingivalis*, *Prevotella intermedia*, *Aggregatibacter actinomycetemcomitans*, *Fusobacterium nucleatum*, *Tannerella forsythia*, *Streptococcus* spp., *Staphylococcus* spp., and *Enterococcus faecalis*	ALAD 5%	PPD, BOP, MOB, REC, and VAS

PPD—probing pocket depth; BOP—bleeding on probing; MOB—mobility; VAS—visual analogue scale; REC—recession.

**Table 8:** Main outcomes and study groups from the RCT.

**Author/Year**	**Study Groups**	**Main Results**
Qamar et al., 2023 [37]	Riboflavin-loaded nanoparticles in aloe vera gel (PGA/RF/AV) + MD. Treated with riboflavin-mediated PDT combined with MD. Treated with MD alone.	Riboflavin-loaded nanoparticles incorporated into aloe vera gel (PGA/RF/AV) both showed effectiveness in treating peri-implantitis in diabetic patients, with the PGA/RF/AV complex achieving superior clinical and microbiological outcomes. While both treatment approaches significantly reduced microbial counts of *Porphyromonas gingivalis* and *Tannerella forsythia* and improved parameters like probing depth, plaque index, and marginal bone levels, the PGA/RF/AV complex was particularly effective in reducing bleeding on probing and minimizing microbial loads over six months. This highlights the potential of combining riboflavin with aloe vera for enhanced antimicrobial and anti-inflammatory effects.

PGA—poly(γ-glutamic acid); RF—riboflavin; AV—aloe vera; MD—mechanical debridement; PDT—photodynamic therapy; PD—probing depth; PI—plaque index; BOP—bleeding on probing.

**Table 9:** Main outcomes and study groups for non-RCT studies.

Reference	Author/Year	Study Groups	Main Results
[39]	Etemadi et al., 2023	Negative control: sterile PBS wash only. Positive Control: 0.12% chlorhexidine. Curcumin alone: moderate antibacterial effect, comparable to PDT with curcumin. Riboflavin alone: lesser antibacterial effect than curcumin. LED alone: minimal effect on CFU/mL reduction. PDT with curcumin + LED: highly effective, second only to chlorhexidine. PDT with riboflavin + LED: moderately effective but less than curcumin-based PDT.	PDT with curcumin + LED was significantly more effective than PDT with riboflavin + LED, showing greater reductions in bacterial colonies (CFU/mL) compared to riboflavin-based PDT, LED alone, and the negative control. Curcumin-based PDT exhibited antimicrobial properties comparable to CHX, the gold standard, highlighting its potential as a non-invasive disinfection method. In contrast, PDT with riboflavin + LED achieved moderate reductions in CFU/mL, though it was less effective than curcumin-based PDT. These results suggest that curcumin is a more promising photosensitizer for PDT due to its superior bactericidal activity and potential as a safe alternative for implant surface decontamination.
[40]	Leelanarathiwatat et al., 2020	Control group Light groups: biofilm exposed to light sources only. Red diode laser: applied for 60 s. Blue LED: applied for 10 s. Photosensitizer groups: biofilm treated with photosensitizers only. Methylene blue: no light activation. FMN: no light activation. Photoactivated PS groups (aPDT): MB with a red diode laser (MB*R): activated for 60 s. FMN with blue LED (FMN*B): activated for 10 s.	Riboflavin-mediated photodynamic therapy using FMN activated by blue LED light demonstrated significant antibacterial effects against Staphylococcus aureus biofilms, achieving a log reduction of 1.23 (approximately 93% reduction in viable bacteria) and a 52–62% decrease in biofilm mass. The blue LED alone showed no antibacterial activity, confirming the essential role of FMN activation. FMN-mediated PDT was as effective as MB-mediated PDT activated by a red diode laser but required only 10 s of irradiation compared to 60 s for MB, offering a faster and more practical treatment option. Additionally, FMN’s light yellow color was noted for cosmetic ease of removal. However, while effective, FMN-PDT did not completely eradicate bacteria, particularly in deeper biofilm layers, suggesting it is best used in combination with other therapies. These findings highlight FMN-PDT as a promising and efficient method for biofilm reduction with potential clinical advantages.
[41]	Mahdizade-Ari et al., 2019	Whole bacterial cell suspension from *A. actinomycetemcomitans* treated with Cur-aPDT. Whole bacterial cell suspension from untreated *A. actinomycetemcomitans*. Cell-free supernatant fluid from *A. actinomycetemcomitans* treated with Cur-aPDT. Cell-free supernatant fluid from untreated *A. actinomycetemcomitans*. Bacterial cell pellet from *A. actinomycetemcomitans* treated with Cur-aPDT. Bacterial cell pellet from untreated *A. actinomycetemcomitans*.	Cur-aPDT significantly reduces microbial cell survival, metabolic activity, and QS abilities in *Aggregatibacter actinomycetemcomitans*, with bystander effects playing a critical role. The therapy decreased cell survival by up to 82.7%, metabolic activity by 42.6%, and QS mediator production by 83.2%, particularly when using treated whole bacterial cell suspensions and cell-free supernatant fluids. These effects suggest that Cur-aPDT generates metabolites with antimicrobial properties that influence neighboring untreated cells, thereby enhancing the overall efficacy of the treatment. However, the therapy showed no significant impact on bacterial efflux pump activity. These findings indicate that the combined direct and bystander effects of Cur-aPDT could make it a potent adjunct therapy for managing localized infections such as periodontitis and peri-implantitis.
[42]	Morelato et al., 2022	Negative control: no surface treatment was applied to the implants. Positive control: surface treatment with 0.2% CHX using a sterile cotton pellet for 60 s. Photodynamic therapy group 1: treatment using a 660 nm diode laser (red light) with 0.1% methylene blue as the photosensitizer. Photodynamic therapy group 2: Treatment using a 445 nm diode laser (blue light) with 0.1% riboflavin as the photosensitizer.	Riboflavin-mediated photodynamic therapy using a 445 nm diode laser for disinfecting contaminated dental implant surface was evaluated against a conventional PDT protocol involving methylene blue with a 660 nm laser and CHX treatment. Results revealed that both PDT approaches significantly reduced Staphylococcus aureus and Candida albicans biofilms, showing comparable efficacy to CHX. The riboflavin-445 nm combination offered aesthetic advantages over methylene blue, as it did not cause discoloration. However, no approach achieved complete microbial eradication, highlighting PDT as a complementary method to mechanical cleaning for peri-implantitis treatment. This study suggests the potential of riboflavin-based PDT as a safe, effective adjunctive therapy, particularly suitable for the aesthetic zone. Further in vivo studies are needed to validate these findings.
[43]	Pourhajibagher et al., 2019	Biofilms treated with GQD. Biofilms treated with curcumin. Biofilms treated with GQD-Cur. Biofilms exposed to blue LED. Biofilms treated with GQD and exposed to blue LED. Biofilms treated with curcumin and exposed to blue LED. Biofilms treated with GQD-Cur and exposed to blue LED. Control: untreated biofilms.	Cur-aPDT using GQD-Cur effectively suppresses perio-pathogens in both planktonic and biofilm forms. GQD-Cur composites were successfully synthesized and characterized using various techniques, confirming their structural and chemical properties, with minimal cytotoxicity to human gingival fibroblasts. Under blue LED irradiation, photoexcited GQD-Cur significantly reduced bacterial viability (93%) and biofilm formation capacity (76%), showing superior antimicrobial effects compared to other treatment groups. The treatment also induced a concentration-dependent increase in ROS production and markedly downregulated key biofilm-related gene expressions, including rcpA, fimA, and inpA, by 8.1-, 9.6-, and 11.8-fold, respectively. These findings highlight GQD-Cur as a promising, non-cytotoxic, nanoscale platform for enhanced antimicrobial photodynamic therapy in periodontitis management.
[44]	Tonon et al., 2022	Curcumin (free): with light activation (L+); without light activation (L−). Curcumin-loaded nanoparticles (Curcumin-NP): with light activation (L+); without light activation (L−). Controls: positive control: 0.12% CHX; negative control: ultrapure water (L−). Nanoparticles without curcumin (NP L+ and L−). 10% DMSO.	Curcumin-NP were successfully synthesized, showing stability, homogeneity, a size of 189 nm, and a 67.5% encapsulation efficiency. Curcumin-NP demonstrated enhanced antimicrobial effects when activated by blue light, particularly against planktonic cultures of *Porphyromonas gingivalis*, *Fusobacterium nucleatum*, and *Streptococcus oralis* but showed limited efficacy against mature multispecies biofilms on titanium surfaces. Blue-light activation improved the photodynamic effects, with curcumin-NP exhibiting higher activity against Gram-negative bacteria like *P. gingivalis* and *F. nucleatum* compared to Gram-positive *S. oralis*. Importantly, the curcumin-NP and free curcumin were non-cytotoxic to human periodontal ligament fibroblast cells. These findings suggest that while curcumin-NP shows promise in antimicrobial PDT, further improvements in biofilm penetration and activity are needed to optimize its therapeutic potential.
[45]	Wenzler et al., 2024	PDT: HELBO^®^ Blue Photosensitizer + laser application. PDT dye: HELBO^®^ Blue. Photosensitizer without laser application. Curcumin/DMSO + laser. Curcumin/DMSO only. DMSO: DMSO solution only, without laser application. PTT: EmunDo^®^ dye + 810 nm laser irradiation. PTT Dye: EmunDo^®^ dye without laser application. Control: untreated group.	Cur-aPDT using 445 nm laser irradiation did not show significant antibacterial improvement compared to curcumin/DMSO without laser activation, suggesting the antibacterial effect was primarily due to the solvent DMSO. DMSO alone demonstrated significant bacterial reduction, highlighting its inherent antimicrobial properties, though its efficacy was slightly reduced when combined with curcumin, possibly due to dilution effects. Compared to conventional PDT or PTT, curcumin-based PDT with 445 nm laser irradiation was less effective, potentially due to suboptimal absorption at this wavelength, indicating a need for further optimization of laser parameters, solvents, and curcumin concentrations.
[46]	Rossi et al., 2022	Periodontitis group (*n* = 10) and peri-implantitis group (*n* = 10)	PDT using 5-ALA demonstrated significant clinical benefits in the treatment of periodontitis and peri-implantitis. A retrospective analysis of 20 patients revealed that adjunctive PDT with 5% 5-ALA gel significantly reduced probing PPD and BOP at 3 and 6 months post-treatment. In periodontal sites, PPD showed a significant reduction (*p* < 0.001), while BOP also decreased significantly (*p* = 0.001). Similarly, in peri-implantitis cases, PPD reduction was statistically significant (*p* < 0.001), though other improvements, such as decreased BOP and slight increases in exposed implant threads, were noted without reaching statistical significance. Patients reported no pain and perceived sustained benefits. These findings support 5-ALA PDT as a promising adjunct to non-surgical therapy for managing periodontal and peri-implant infections
[47]	Petrini et al., 2022	MACHINED (control group), MACHINED + ALAD (experimental group), and DAE (control group)	PDT using 5-ALA demonstrated significant antibacterial and antibiofilm activity against *Streptococcus oralis* biofilm on titanium implant surfaces. The study showed that applying a 5% 5-ALA gel followed by red LED light irradiation (630 nm) significantly reduced bacterial CFUs and biofilm biomass on both machined and DAE titanium surfaces. CFU counts decreased by 89% on machined surfaces and 77% on DAE surfaces compared to unexposed controls (*p* < 0.050). Live/dead staining confirmed 100% bacterial cell death in PDT-treated samples, and SEM revealed a significant reduction in biofilm accumulation. These findings support 5-ALA-mediated PDT as an effective strategy for implant decontamination and peri-implant disease management.
[48]	Radunović et al., 2020	*Enterococcus faecalis* (1-h incubation with 10% and 50% ALAD); *Enterococcus faecalis* (25 min incubation with 10%, 25%, and 50% ALAD); *Escherichia coli* (25 min incubation with 10%, 25%, and 50% ALAD); *Staphylococcus aureus* (25 min incubation with 10%, 25%, and 50% ALAD); *Veillonella parvula* (25 min incubation with 10%, 25%, and 50% ALAD); *Porphyromonas gingivalis* (25 min incubation with 10%, 25%, and 50% ALAD).	PDT using ALAD gel and red LED irradiation demonstrated a significant antibacterial effect across all tested bacterial species. Enterococcus faecalis showed total inactivation with 50% ALAD + 7 min LED, while lower ALAD concentrations (25%) combined with 5 min LED were effective in reducing CFUs. Escherichia coli and *Porphyromonas gingivalis* required 50% ALAD for significant reduction, with LED irradiation enhancing bacterial eradication at lower concentrations. Staphylococcus aureus and *Veillonella parvula* exhibited intrinsic sensitivity to ALAD alone, but LED irradiation amplified the bactericidal effect. Overall, 25 min of 50% ALAD incubation followed by 5 min LED irradiation was the most effective protocol, achieving substantial bacterial reduction, with implications for treating oral infections and antibiotic-resistant bacteria.

PBS—phosphate-buffered saline; CFU/mL—colony-forming units per milliliter; PDT—photodynamic therapy; LED—light-emitting diode; PS—photosensitizer; MB—methylene blue; FMN—flavin mononucleotide); aPDT—antimicrobial photodynamic therapy; WBCST—whole bacterial cell suspension treated; WBCSU—whole bacterial cell suspension untreated; CFSFT—cell-free supernatant fluid treated; CFSFU—cell-free supernatant fluid untreated; BCPT—bacterial cell pellet treated; BCPU—bacterial cell pellet untreated; QS—quorum sensing; CHX—chlorhexidine; GQD—graphene quantum dots; GQD-Cur—graphene quantum dots with curcumin; ROS—reactive oxygen species; PGA/RF/AV—poly(glycolic acid)/riboflavin/aloe vera; MD—mechanical debridement; NP—nanoparticles; DMSO—dimethyl sulfoxide; HELBO^®^—a specific brand of Blue Photosensitizer for PDT; PTT—photothermal therapy; Curcumin-NP—curcumin-loaded polymeric nanoparticles; Cur-aPDT—curcumin-mediated antimicrobial photodynamic therapy; DAE—double-acid-etched; ALAD—5-aminolevulinic acid.

**Table 10:** Light sources physical parameters of the RCT that fulfilled the eligibility criteria.

Author/Year	Light Source	Operating Mode	Wavelength (nm)	Energy Density (J/cm^2^)	Power Output (mW)	Powermeter Used	Energy Output (J)	Irradiation Time (s)	Power Density (mW/cm^2^)
Qamar et al., 2023 [37]	Diode laser (670 nm)	Continuous wave	670	1.1	280	Not specified	Not specified	60	1100

**Table 11:** Light sources physical parameters of studies that fulfilled the eligibility criteria.

Author/Year	Light Source	Operating Mode	Wavelength (nm)	Energy Density (J/cm^2^)	Power Output (mW)	Powermeter Used	Energy Output (J)	Irradiation Time (s)	Power Density (mW/cm^2^)
Etemadi et al., 2023 [39]	LED (DY400-4, Denjoy, China)	Continuous wave	390–480	300–420	1000 ± 100	LaserPoint s.r.l, Milano, Italy		300	
Leelanarathiwatat et al., 2020 [40]	Blue LED and red diode laser	Pulsed wave	450–470, 670	37–40, 4.24	291–314 12	Not specified	29.1–31.4 0.2	10 60	3700–4000 75
Mahdizade-Ari et al., 2019 [41]	Blue LED	Continuous wave	435 ± 20	300–420	Not specified	Not specified		300	1000–1400
Morelato et al., 2022 [42]	Diode laser	660 nm laser: Continuous-wave mode 445 nm laser: Pulsed mode (100 Hz)	445 660	1.24 1240	100	Not specified	1.24	60	124.34
Pourhajibagher et al., 2019 [43]	Blue LED	Continuous wave	60–420	Not specified	1000–1400	LaserPoint s.r.l, Milan	Not specified	60	1000–1400
Tonon et al., 2022 [44]	Blue Light	Fixed output power	420	72	-	Not specified	Not specified	720	95.5
Wenzler et al., 2024 [45]	HELBO^®^ TheraLite laser SIROLaserBlue FOX Q810plus laser	Continuous wave	660 445 810	Not specified	100 600 200	Not specified	Not specified	10 (per point × 6)	70.74 373.02 565.90
Petrini et al., 2022 [47]	AlGaAs power LED device (TL-01)	Not specified	630 ± 10	100	380	Not specified	Not specified	420	380
Radunović et al., 2020 [48]	AlGaAs power LED (TL-01)	Continuous	630 ± 10	23	Not specified	Not specified	Not specified	Not specified	380

LED—light-emitting diode.

## Data Availability

Not applicable.
